# From silos to synergy: a consortium approach to air pollution and public health in Abu Dhabi

**DOI:** 10.1186/s41256-024-00383-w

**Published:** 2024-10-05

**Authors:** Barrak Alahmad, Ernani F. Choma, Basem Al-Omari, Eman Alefishat, Abdu Adem, John S. Evans, Petros Koutrakis, Senthil Rajasekaran

**Affiliations:** 1 Environmental Health Department, Harvard T.H. Chan School of Public Health, 401 Park Dr, 4th floor, Boston, MA 02115 USA; 2 College of Medicine and Health Sciences, Khalifa University of Science and Technology, PO Box 127788, Abu Dhabi, UAE; 3 JS Evans and Associates, Consultants in Environmental Health, Cary, NC USA

**Keywords:** PM2.5, Middle East, Gulf Countries, Risk Uncertainty, Decision-making

## Abstract

Financial resources alone cannot guarantee effective public health policy. In Abu Dhabi, massive economic growth in the desert climate resulted in concentrated urbanization and led to challenges in the regulation of air pollution. The Environment Agency in Abu Dhabi commissioned us to scope the regulatory challenges for air pollution. Part of this project relied on the participation and involvement of key stakeholders. We found three barriers: (1) limited appreciation of uncertainties in risk estimates and discussion on the importance of considering control costs and the societal trade-offs between health and wealth inherent in such decisions, (2) compartmentalization of efforts, and (3) challenges to decide how to prioritize risks in policy agendas. We propose a consortium-like approach that brings stakeholders together and places risk, uncertainty, and tradeoffs between health and wealth at the forefront of decision-making. Expected outcomes include improved collaboration and information sharing, strategic prioritization of emission controls, and a better understanding and consideration of uncertainty to guide future public health research.

## Background

In the southeastern part of the Arabian Peninsula, Abu Dhabi is known for its arid desert environment and extreme heat. Since the establishment of the United Arab Emirates (UAE), Abu Dhabi has experienced rapid economic growth followed by significant urbanization and industrial development. As a growing economy, Abu Dhabi faces an environmental health challenge in regulating air pollution due to the presence of dust storms, emissions from the petrochemical industry, and vehicles [1, 2]. Air pollution is associated with increased mortality rates and higher morbidity, leading to more absenteeism from school and work and greater demands on the healthcare system. While local studies in Abu Dhabi have not examined all these impacts (except for mortality in 2008 [3]), similar patterns have been observed globally where comprehensive studies have been conducted. In response to these challenges, a collaborative effort was formed following a call by the Environment Agency—Abu Dhabi (EAD) to scope the impact of environmental pollutants on human health and identify barriers to policymaking. This policy brief presents lessons learned from this collaboration and provides an outlook on efforts to regulate air pollution in Abu Dhabi.

## A stakeholder workshop

In December 2023, a stakeholder workshop in Abu Dhabi marked a significant milestone. We selected pivotal stakeholders representing diverse sectors to work together to refine air pollution research and regulatory priorities. The stakeholder group included academics, key representatives from the Department of Health, the public health centers, the healthcare centers, and the environmental agencies. In view of the tight project schedule, pivotal stakeholders were identified and selected based on recommendations from Khalifa University and the EAD. Future efforts could consider more formal and systematic approaches of stakeholder selection.

The feedback and insights gathered during this workshop were invaluable in refining research priorities and ensuring that the future roadmap aligns with the collective goal of improving air quality and public health in Abu Dhabi.

## Identified barriers to policymaking

We found three barriers: (1) the uncertainty in risk estimates and the benefits of acknowledging and quantifying those uncertainties, as well as a discussion of the importance of estimating the costs for potential controls and the role of societal tradeoffs between health and wealth inherent in any such decisions, (2) compartmentalization of efforts and working in silos, and (3) challenges to decide how to prioritize risks in policy agendas.

### Risk, uncertainty, and societal values

The focus of the workshop was on risk management in environmental health, with the discussion driven towards regulating air pollution. A central focus was the dilemma of deciding what exposure controls to prioritize, acknowledging that science provides essential data and insights but cannot determine ‘acceptable levels of risk’, and that decisions often involve complex trade-offs, weighing the potential health benefits against economic, social, and political factors. Furthermore, many stakeholders involved in such decisions are unlikely to hold similar views about values and often have very different ideas about the inevitable tradeoffs between health and wealth. Differences in values are best brought into the open and recognized as legitimate elements of societal decision making.

An additional challenge is that this decision-making involves substantial uncertainty about the magnitude of risks and the health benefits of emission control policies. This uncertainty stems from fundamental limitations in scientific understanding of both the biology and toxicology underlying human disease development and in determining the impacts of specific emissions sources on human exposure to environmental contaminants. Decision-makers typically hold different preferences for risk—some may be risk averse, whereas others may be risk neutral or risk seeking. Recognizing these potential differences in attitudes towards uncertainty offers real potential to disentangle issues of science from issues of value and to improve the clarity and efficiency of decision and policy making.

To navigate some of these challenges, our approach emphasized: (i) the importance of incorporating uncertainty into the decision-making process and conducting value-of-information analyses to help plan future research aimed at reducing this uncertainty; and (ii) the benefits of recognizing the distinct and important role of societal values in decision-making, to avoid falling into the ‘let science speak’ trap—pretending that science itself can resolve complex public policy dilemmas that have economic costs, and other social consequences of policies, and ignoring tradeoffs between health benefits and economic costs or other consequences.

**Fig. 1:**
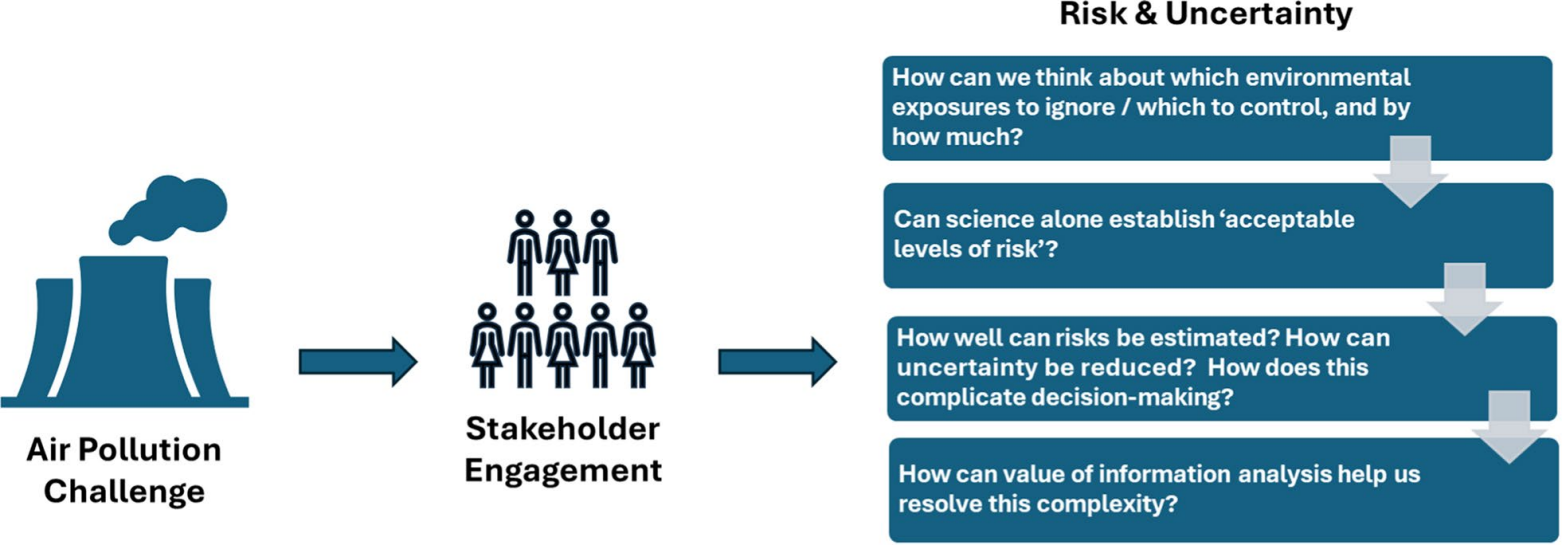
Process to decisions, risk, and uncertainty

Structured expert judgment [4] is necessary to characterize the uncertainty about fine particulate matter (particulate matter with aerodynamic diameter less than 2.5 microns, PM_2.5_) health effects in Abu Dhabi due to lack of local studies and to the complexity of the issues involved in borrowing literature from the United States, Europe, and other countries, to apply to the Middle East, such as the possibility of differential toxicity of PM_2.5_ components, the multiplicity of possible concentration–response functions at high PM_2.5_ levels, among others [5]. The value of information analysis then plays a crucial role, using uncertainty to help determine which additional data and research can be most valuable, by considering whether the cost of research (financial or otherwise) justifies the added value it may provide in the form of improved decision-making due to reduced uncertainty [6]. This approach enables a more strategic allocation of resources. Ultimately, by placing these considerations of risk and uncertainty front and center in our discussion, we set the stage for more nuanced and effective decision-making processes (Fig. 1).

### Challenges of working in silos

We then found compartmentalization of efforts that were unintentional in monitoring and researching air pollution in the UAE. There were commendable initiatives led by each of the stakeholder groups. Since 2007, Abu Dhabi built an extensive network of 20 fixed and 2 mobile stations for measuring particulate pollution, accumulating over 1.5 billion valid minute-data points. The Emirate also commissioned innovative endeavors like remote sensing of vehicle emissions and ship monitoring. Nevertheless, the integration of this data into policymaking was requiring action. Similarly, detailed environmental health risk assessment work conducted in the past decade [3] has not been effectively synthesized nor translated into broader policy measures. Again, the root cause appears to be the siloed nature of the organization of these efforts, with those responsible for pollution monitoring, health effects analysis, and policy development working separately and largely independently of each other. Much of the crucial data and insights remain isolated within specific entities. These problems are not unique to Abu Dhabi or the UAE, but are common throughout the region and across the globe. This disconnect hinders the creation of comprehensive regulatory standards, such as for PM_2.5_, and limits the impact of these valuable datasets and assessments on policy formulation and implementation.

### Prioritization for policymaking

In our discussion of the barriers to effective policymaking, the main criterion that emerged regarding the prioritization of environmental exposures for control was their potential impact on public health. Specifically, the focus was drawn to air pollution, notably PM_2.5_, as a priority concern due to its high impact on public health as compared to other pollutants, such as those commonly found in contaminated soil. This prioritization recognizes the substantial body of evidence linking PM_2.5_ exposure to large effects on a range of adverse health outcomes, making it an immediate and substantial threat to public health [7]. The decision-making process, therefore, emphasized targeting those exposures that promise the highest yield in terms of health benefits.

## Transitioning from silos to collective efforts

A proposed consortium-like approach was applied, focusing on collaborative efforts among stakeholders in Abu Dhabi. During our stakeholder workshop, a consensus emerged that the Environmental Agency, academic researchers, as well as the Department of Health, were largely unaware of essential efforts undertaken by the other parties. Our approach effectively breaks down existing silos to create synergies in air pollution management, extending from exposure assessment and epidemiology to risk assessment and management.

Our proposed synergistic collaborative approach puts forward the following:

- *Collaboration and information sharing among stakeholders* Facilitate the exchange of efforts, ideas, data, and methodologies among local experts and institutions, and devote effort to development of shared goals and measures of success—with an eye toward identifying policies which can substantially reduce exposures with large public health impacts, while doing this in a cost-effective or cost-beneficial manner—in order to craft solutions tailored to specific local needs. The Open Risk Assessment by Tuomisto and Pohjola [8] offers an example of open mass collaboration in risk assessment work.
- *Strategic prioritization of emission controls and other exposure reduction policies* Focus efforts on controlling and reducing exposure to pollutants with large impacts on public health. Ideally, these would be identified through preliminary risk assessments conducted for the largest possible set of pollutants. For the small set of pollutants identified as important, the benefits of various possible emission controls could be estimated and weighed against control costs (financial or otherwise). This second, more sophisticated, analysis would include assessment of uncertainty and would facilitate identification of the most cost-beneficial policies. The work of MacDonald-Gibson [3] for Abu Dhabi and the UAE provides an excellent illustration of the benefits of phased screening analyses and indicates that in the UAE, air pollution—particularly from PM_2.5_—deserves further exploration to determine the costs and benefits of various possible controls. Our recommended approach is rooted in Morgan and Henrion’s [9] advice of iteratively refining the analysis and in the wisdom of Finkel and Golding [10] that we should be guided by the cost-effectiveness of controls rather than focusing on large impacts, without regard to cost.
- *Acknowledgements of uncertainty and strategic research plan* Embrace the complexity of environmental health issues and the uncertainty in estimates of risks associated with exposure to pollutants and of health benefits of source controls. Additional research can reduce this uncertainty, and strategic research plans using formal value of information analyses can help identify which studies should be prioritized.

Tackling public health challenges is quite complex. Providing financial resources alone (without attention to organizational structure, information flows and development of shared goals and measures of success) is not enough to guarantee effective public health policy. Policymakers should establish clear communication channels and allocate resources strategically to ensure the success of this collaborative effort. Embracing change will be essential to achieving meaningful improvements in air pollution management and public health outcomes.

## Data Availability

Not applicable.

## Abbreviations

UAE: United Arab Emirates
EAD: Environmental Agency—Abu Dhabi
PM_2.5_: Particulate matter with aerodynamic diameter less than 2.5 µm
